# Gene Expression Signatures Identify Biologically Homogenous Subgroups of Grade 2 Meningiomas

**DOI:** 10.3389/fonc.2020.541928

**Published:** 2020-11-05

**Authors:** Zsolt Zador, Alexander P. Landry, Ashirbani Saha, Michael D. Cusimano

**Affiliations:** Division of Neurosurgery, Department of Surgery, St. Michael’s Hospital, Toronto, ON, Canada

**Keywords:** meningioma, transcriptomics, gene expression networks, bioinformatics, marker discovery

## Abstract

**Introduction:**

Meningiomas are the most common brain tumor, with prevalence of approximately 3%. Histological grading has a major role in determining treatment choice and predicting outcome. While indolent grade 1 and aggressive grade 3 meningiomas exhibit relatively homogeneous clinical behavior, grade 2 meningiomas are far more heterogeneous, making outcome prediction challenging. We hypothesized two subgroups of grade 2 meningiomas which biologically resemble either World Health Organization (WHO) grade 1 or WHO grade 3. Our aim was to establish gene expression signatures that separate grade 2 meningiomas into two homogeneous subgroups: a more indolent subtype genetically resembling grade 1 and a more aggressive subtype resembling grade 3.

**Methods:**

We carried out an observational meta-analysis on 212 meningiomas from six distinct studies retrieved from the open-access platform *Gene Expression Omnibus.* Microarray data was analyzed with systems-level gene co-expression network analysis. Fuzzy C-means clustering was employed to reclassify 34 of the 46 grade 2 meningiomas (74%) into a benign “grade 1-like” (13/46), and malignant “grade 3-like” (21/46) subgroup based on transcriptomic profiles. We verified shared biology between matching subgroups based on meta-gene expression and recurrence rates. These results were validated further using an independent RNA-seq dataset with 160 meningiomas, with similar results.

**Results:**

Recurrence rates of “grade 1-like” and “grade 3- like” tumors were 0 and 75%, respectively, statistically similar to recurrence rates of grade 1 (17%) and 3 (85%). We also found overlapping biological processes of new subgroups with their adjacent grades 1 and 3.

**Conclusion:**

These results underpin molecular signatures as complements to histological grading systems. They may help reshape prediction, follow-up planning, treatment decisions and recruitment protocols for future and ongoing clinical trials.

## Background

Meningiomas are the most common adult brain tumor, carrying an overall prevalence of approximately 3% in the population (1, 2). Histopathologic analysis is the mainstay of diagnosis and, together with the extent of surgical resection, is a key determinant of outcome and treatment planning (3, 4). According to World Health Organization (WHO) grading, the majority of meningiomas (almost 70%) constitute grade 1, of which about two thirds are cured with surgical excision alone (4) and 15–20% recur within 5 years of diagnosis (5–8). Grade 3 meningiomas, by contrast, are rare and aggressive with a 5 year recurrence rate of approximately 90% (4). These extremes of histological grades have relatively homogenous clinical behavior, yet grade 2 histopathologic variants, which constitute 20–30% of all meningiomas, represent a biological intermediate. Predicting the clinical course and treatment response for these tumors is particularly challenging (9) given their heterogeneous biology, and the 5-year recurrence rate of grade 2 meningiomas is approximately 50% (10–12). This uncertainty is corroborated by regular revisions in WHO definitions (13), overlapping molecular signatures with adjacent grades (14, 15) and open questions about the benefits of chemotherapy (9, 16) and adjuvant radiation (17–19) for these tumors. Defining subgroups of grade 2 meningiomas with homogenous biological and clinical properties may be critical to successfully resolving these questions, thereby improving prognostication and treatment for patients.

Molecular markers have been successfully implemented in heterogenous diseases like glioblastoma (20) and medulloblastoma (21) to identify subgroups with shared biology and clinical outcome. Several studies have also examined markers for meningioma biology (14, 22, 23). These previous findings suggest that some grade 2 meningiomas share features with grade 1s while others more closely resemble grade 3s based on clinical behavior and genetic features such as somatic mutations, copy number variants (15, 24, 25), methylation status (14), and genome wide expression profiles (22, 26). Most research on gene expression in meningioma, however, focuses on single-gene analytics. This is not optimized for the low and additive molecular signals which frequently underlie complex and heterogeneous diseases. Systems biology approaches such as co-expression networks (27, 28), on the other hand, are able to provide a higher resolution of these complex genetic processes (27, 29–31).

In this study we hypothesize that grade 2 meningiomas can be segregated into homogeneous subgroups that either resemble indolent grade 1 tumors or aggressive grade 3s. Our aim was to establish gene expression signatures using co-expression networks to identify homogenous subgroups of grade 2 meningiomas.

## Materials and Methods

This study is an observational analysis of open-source data from the repository *Gene Expression Omnibus* (GEO) (32) and therefore does not require IRB review. All studies with human meningioma microarray data annotated with WHO grade were included in the analysis, which yielded six studies. Another study using RNA-seq transcriptomics constituted an external validation cohort (Table 1). All studies included in our meta-analysis were published after the 2007 edition of WHO grading for meningiomas, suggesting this classification was implemented in these studies. For each study, the data was backgrounded corrected, quantile normalized, and log-2 transformed using the *Affy* (33) and *Limma* (34) R (The R Project) packages for Affymetrix and Illumina/RNA-seq platforms, respectively. After selecting only the genes which were common to the six microarray studies, the studies were merged, scaled to a global mean and standard deviation of 0 and 1, respectively (35), and batch-corrected using *ComBat*, a well-established empirical Bayes approach (36). The same approach was used to batch-correct the RNA-seq study, which had been divided into “Discovery” and “Validation” cohorts. The resultant data matrices were used during all subsequent analysis.

**TABLE 1:** Study demographics.

**GEO entry**	**Platform**	***N***	**Mean age (SD)**	***N* male (%)**	**WHO grade (*n*)**			**N recurrence (%)**	**Median F/U^1^ (95% CI)**	**Median TTR^2^ (95% CI)**
					**I**	**II**	**III**			
GSE100534	GPL6244	8	N/A	3 (37.5)	6	1	1	N/A	N/A	N/A
GSE77259	GPL6244	14	54.1 (10.1)	4 (28.6)	10	4	0	N/A	N/A	N/A
GSE54934	GPL6244	22	N/A	N/A	20	2	0	N/A	N/A	N/A
GSE43290	GPL96	47	61.7 (15.0)	13 (27.7)	33	12	2	8 (17.0)	4.7 (3.7–5.7)	5.8 (3.6–8.0)
GSE16581	GPL570	68	63.2 (14.7)	25 (36.8)	43	19	6	13 (56.5)	4.7 (4.1–5.3)^3^	N/A
GSE74385	GPL10558	53	N/A	N/A	17	8	28	22 (48.9)	N/A^4^	N/A
Overall^5^		212	61.7 (14.6)	45 (32.8)	129	46	37	43 (37.4)	4.7 (4.3–5.4)	N/A
GSE136661	Illumina HiSeq400	145	58.0 (13.5)	52 (35.9)	116	29	0	22 (15.1)	N/A^6^	N/A

*^1^Follow-up (years). ^2^Time to recurrence (years). ^3^Time to survival (time until death or end of study). ^4^Follow-up at least 3 years for non-recurrent tumors, though specific times are not available. ^5^Of available data. ^6^Follow-up reported as 0–91 months (up to 7.6 years) with a median of 28 months (2.3 years), though specific times are not available.*

Differential gene expression analysis was used to compare grades 1 and 3 meningiomas. In log2-transformed space, the fold change (FC) was computed by subtracting the mean expressions of each gene in grade 1 tumors from the corresponding mean expressions in grade 3 tumors. Genes with absolute log2-transformed FC ≥ 1.5 and *p* ≤ 0.0001 were considered significant.

We used the well-established “Weighted Gene Correlation Network Analysis” (WGCNA) to detect “modules” (clusters) of strongly co-expressed genes (29). Per these previously described techniques, we first computed an “adjacency matrix” using soft-thresholded Pearson correlations between each gene pair. This was converted into a biologically-inspired topological overlap map (TOM), wherein pairwise gene similarities are derived from comparing their connectivity profiles (37). Hierarchical clustering converted the TOM into a dendrogram, and a subsequent “dynamic” tree-cut (38) served to identify gene modules. These modules were annotated the annotation platform *Enrichr* (39), an open-source bioinformatics resource. Additionally, representative module “meta-genes” for each sample were computed as the first principal component of their constituent genes’ expression values. The utility of this approach was verified in our dataset by demonstrating that higher principle components capture a very small proportion of the overall variance (Supplementary Figure S1A) and showing that neither study batch nor sex cluster along the first principle component (Supplementary Figures S1B,C). This eliminates the possibility of batch effect or sex being drivers of our “meta-gene” values and confounding results. Differences in the expression levels of these “meta-gene” between grades was tested with a Mann–Whitney test, with a *p* ≤ 0.05 considered significant.

In order to better understand the heterogeneity of grade 2 meningiomas, we began by identifying genetic signatures able to best distinguish grades 1 and 3 alone. Fuzzy C-means (FCM) clustering was applied to the set of all patients in our study and the resultant separation of grades 1 and 3 was established with a sigmoidal cost function that is balanced for differences in the prevalence of both grades:

$$C=\frac{1}{N_{1}}\,\sum_{i\in S_{1}}\left(\frac{1}{1+e^{-\alpha \,\left(P_{i}-0.5\right)}}\right)+\frac{1}{N_{3}}\,\sum_{j\in S_{3}}\left(\frac{1}{1+e^{-\alpha \,\left(0.5-P_{j}\right)}}\right)$$

In the above equation, *N*_1_ and *N*_3_ are the number of grade 1 and grade 3 tumors, respectively; *S*_1_ and *S*_3_ are the sets of grade 1 and grade 3 tumors, respectively; *P*_*k*_ is the FCM clustering-derived probability of patient *k* being in the grade 3-enriched cluster; and α is a tunable hyperparameter. We used a two centroid model wherein cluster polarity was established by comparing the ratio of grade 3 to grade 1 tumors at both ends of the probability distribution (hard-thresholding at 80% probabilities).

Single genes and module “meta-genes” which were significantly different between grade 1 and grade 3 tumors served as input variables. Backward elimination and forward selection were used for feature selection with model performance measured using the above cost function. Hyperparameter (α) values of 1, 5, 10, and 100 tested for all models. Once the separation of grades 1 and 3 was optimized, the probability distribution of grade 2 meningiomas within the same output was investigated. Grade 2 meningiomas with a probability ≥80% of being in the grade 1-enriched cluster were defined as “grade 1-like,” and those with a probability ≥80% of being in the grade 3-enriched cluster were defined as “grade 3-like.”

We first compared the recurrence rates of “grade 1-like” and “grade 3-like” meningiomas, and compared each to the rates of grade 1 and grade 3 tumors. Notably, only 115 of the 212 patients in our cohort have annotated recurrence, though all had recurrence labels in the RNA-seq validation cohort. To investigate the degree of biological overlap between “grade 1-like” and grade 1 meningiomas, and similarly between “grade 3-like” and grade 3 meningiomas, we used the correlation between their module “meta-gene” expression levels. In addition, we compared the biological separation between the newly described subtypes of grade 2 meningiomas to the separation of grades 1 and 3 by correlating their differential module expression levels.

All computational work relied on the open-source computational platform R (40) (The R Project), including packages *WGCNA* (29), *ppclust* (41), *Affy* (33), *Limma* (34), and *SVA* (42).

### Statistical Methods

Transcriptomic expression levels were analyzed using the two-sample *t*-test and Mann–Whitney U test. Recurrence rates were compared with a Chi-square test. Notably, since only a subset of samples had recurrence annotated, recurrence analysis was only performed on this subset of patients.

## Results

### Participants, Descriptive, and Outcome Data

Please refer to Table 1 for details of our study cohort. In brief, we included six microarray series [GSE100534 (43), GSE77259 (44), GSE54934 (45), GSE43290 (46), GSE16581 (47), GSE74385 (48)] with a combined 212 patients and one RNA-seq series [GSE136661 (49)] with 145 patients. The distribution of histopathologic subtypes are illustrated in Supplementary Figure S2. We identify two subgroups of grade 2 meningiomas with significantly different recurrence rates among those with available data (75% in the aggressive subgroup and 0% in the indolent subgroup, *p* < 0.005). These recurrence rates are similar to the recurrence rates of grades 3 and 1, respectively, suggesting clinical utility in this reclassification. A more detailed outline of our results can be found below.

### Main Results

**FIGURE 1:**
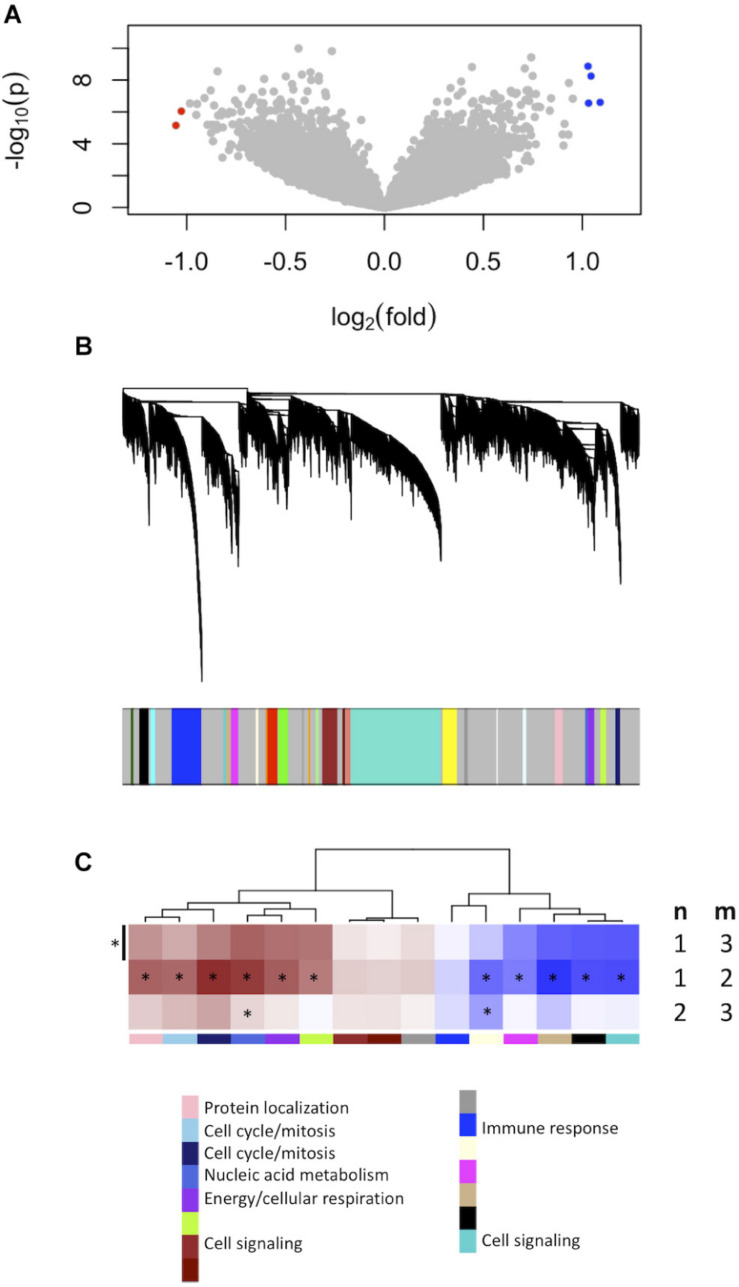
Gene expression signatures associated with meningioma grade. **(A)** Differential gene expression between grades 3 and 1 meningiomas reveal four upregulated and two downregulated genes in grade 3 tumors [| log_2_(fold change)| ≥ 1.5, *p* < 0.0001] highlighted with blue and red dots, respectively. **(B)** Gene co-expression networks analysis. Dendrogram of genes based on the topological overlap map, with the 29 gene modules represented by colors in the bar below. Gray represents unclassified genes. **(C)** Plot of median module meta-gene expression differences between grades (“m” versus “n”). Only modules with significantly different expression between grades 1 and 3 are included (Mann–Whitney *p* < 0.05). Red indicates modules which are upregulated in grade (m), and darker shades indicate larger effect sizes. Notably, 11/15 modules are significantly different between grades 1 and 2 while 2/15 are also significantly different between grades 2 and 3. *p < 0.05 (Mann–Whitney).

We firstly established the gene expression profile that differentiates grade 1 from grade 3 meningiomas. Differential gene expression showed four up-regulated and two down-regulated genes (log_2_ fold change ≥1.5, *p* ≤ 0.0001) summarized in Figure 1A and Supplementary Table S1.

We created another signature to distinguish grade 1 from grade 3 meningiomas using gene co-expression networks. This yielded 29 co-expressed gene modules (Figure 1B), of which 15 had median meta-gene expression levels that differed significantly between grades 1 and 3 (Mann–Whitney *p* < 0.05, Figure 1C). A subset of these 15 were also significant between grades 1 and 2 and/or between grades 2 and 3 tumors, suggesting the intermediate biology of grade 2 meningiomas.

**FIGURE 2:**
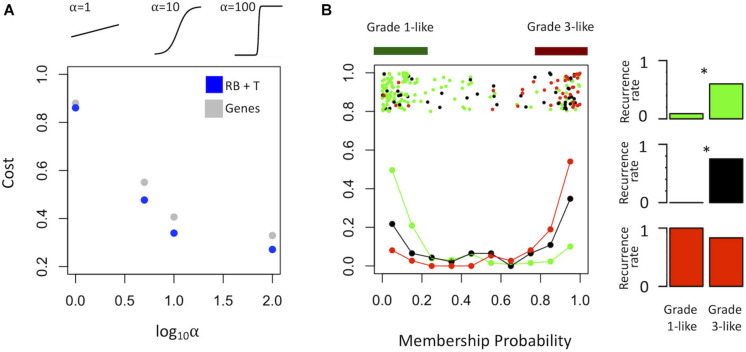
Optimized soft clustering reveals two subgroups of grade 2 meningiomas. **(A)** Cost of multiple input configurations: “royal blue” (RB) and “tan” (T) modules in blue and optimized differentially expressed genes in gray. Top inset depicts shape of sigmoid function with varied alphas. **(B)** Summary graph of fuzzy C-means clustering best performing inputs (RB + T). The *x*-axis represents the probability of being in the grade-3 enriched cluster and *y*-axis represents the proportion of patients in each bin of 10%. Line graph component represents normalized frequency distribution of each histological grade (green = grade 1, black = grade 2, red = grade 3). Top jitter plot represents individual patients. Dark green and red bars above represent the 20 and 80% thresholding into grade 1-like and grade 3-like subgroups of grade 2 meningiomas. Recurrence rates are plotted on the right by grade (green, black, red) and subgroup (“grade 1-like” and “grade 3-like”). *Chi-square *p* < 0.05.

**FIGURE 3:**
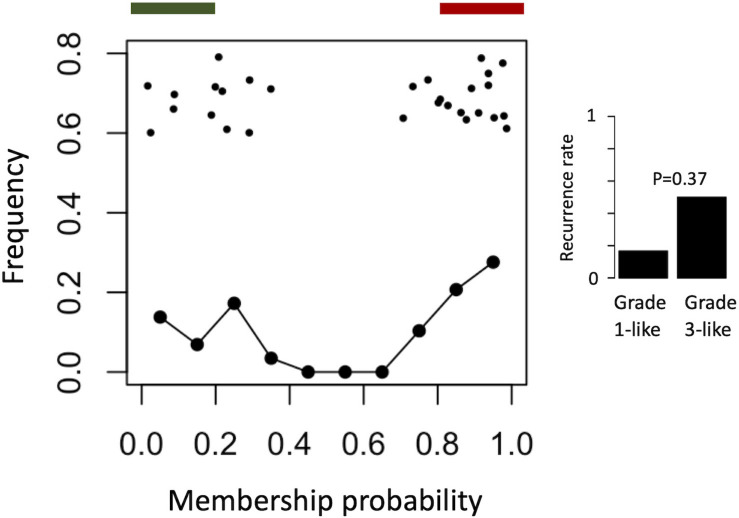
Validation of meningioma reclassification using RNA-seq data. Summary graph of fuzzy C-means clustering best performing inputs on the microarray data (modules RB + T). The *x*-axis represents the probability of being in the grade-3 enriched cluster and *y*-axis represents the proportion of patients in each bin of 10%. Line graph component represents normalized frequency distribution of each histological grade (green = grade 1, black = grade 2, red = grade 3). Top jitter plot represents individual patients. Dark green and red bars above represent the 20 and 80% thresholding into grade 1-like and grade 3-like subgroups of grade 2 meningiomas. Recurrence rates are plotted on the right by grade and subgroup (“grade 1-like” and “grade 3-like”). Notably, recurrence data is not available for the grade 3 meningiomas in this cohort.

To find a genetic signature that best differentiates grades 1 and 3 tumors, we used two-centroid soft clustering and evaluated the resultant distribution of patients with a balanced sigmoidal cost function (with a lower cost being indicative of greater average separation). An iterative feature selection approach was conducted using single genes and gene modules which were differentially expressed between grades 1 from 3. Notably, modules (represented by their meta-gene expression) consistently yielded better performance (lower cost) than single genes (Figure 2A). The lowest cost was achieved with two modules as inputs; one of which contained 61 genes which map predominantly to purine biosynthesis and the other consisted of 121 genes which map strongly to mRNA splicing. Gene lists for both of these modules can be found in the supplemental content. We then used this signature to reclassify grade 2 meningiomas. Importantly, this signature was derived without the clustering model having any input from grade 2 meningiomas during training. Using 80% membership probability as a cutoff, we reclassified 34 of 46 grade 2 meningiomas (74%) into a “grade 1-like” (13/46) and “grade 3-like” (21/46) subgroup of grade 2 meningiomas (Figure 2B). A small group of 12 grade 2 meningiomas did not fall into either “grade 1-like” or “grade 3-like” groups and may therefore represent a true biological intermediate. The histopathologic subtype was annotated for 7/13 “grade 1-like” tumors (six atypical, one transitional with brain invasion) and 7/21 “grade 3-like” tumors (six atypical, one atypical with brain invasion). Of the 12 unclassified grade 2 meningiomas, 2 were atypical, 1 was meningothelial with brain invasion, and 9 were not annotated. Recurrence rates were available for only a subset of cases (30/46) and were significantly higher in “grade 3-like” (9/12) compared to “grade 1-like” (0/8) subgroups (*p* < 0.005). Concordantly, there was no significant difference in recurrence rates between grade 1 and “grade 1-like” groups (10/59 versus 0/8) nor between grade 3 and “grade 3-like” groups (22/26 versus 9/12). Of the 12 unclassified grade 2 meningiomas 2 recurred, 8 had no documented recurrence and 2 had unknown recurrence status. Comparatively, we reclassified 20 of 29 grade 2 meningiomas in the RNA-seq validation cohort (69%) using the same gene signatures and thresholding (6 “grade 1-like” and 14 “grade 3-like”) (Figure 3). The recurrence rates of “grade 3-like” and “grade 1-like” were 1/6 (17%) and 7/14 (50%), respectively. However, the numbers were too small to achieve statistical significance.

**FIGURE 4:**
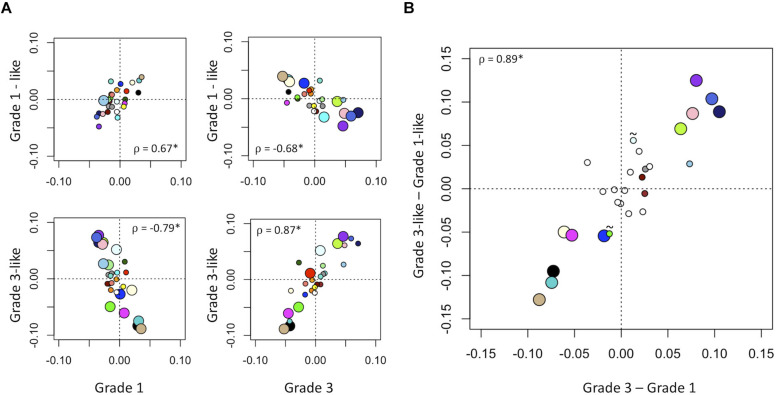
Molecular identity of newly described grade 2 meningioma subgroups. **(A)** Scatter plot of median module meta-gene expression (unitless). Larger circles indicate Mann–Whitney *p* < 0.05. Colors correspond to previously identified modules in Figure 1B. ρ = Pearson coefficient, *p < 0.05. Note the positive correlation between the modules of grade 1 and “grade 1-like” and grade 3 and “grade 3-like” subtypes. **(B)** Scatter plots of genetic separation between grade 2 subtypes as histological grades. The *x*-axis represents the difference in median module expression between grades 3 and 1, while the *y*-axis represents the difference in median module expression between “grade 3-like” and “grade 1-like.” Large circles represent modules which are significantly different in both comparisons and empty circles indicate modules which are not significantly different in either. Of the remainder, 4/6 are significantly different between grades 3 and 1 only and 2/6 is significantly different between “grade 3-like” and “grade 1-like” (∼).

Next, we verified the molecular identity of the newly detected subgroups of grade 2 meningiomas. Using a systematic comparison based on median module expression levels (Figure 4), we found concordance between the biology of our newly identified grade 2 subtypes with their adjacent grade (Figure 4A). Differential analysis also suggested that the overall biological separation between the newly described subgroups is similar to the separation between grades 1 and 3 in module space. These findings further lend to the validity of dividing grade 2 meningiomas into biologically homogenous subgroups which parallel existing grades.

## Discussion

### Key Results

Our study focuses on the most heterogenous group of meningiomas: WHO grade 2. We were able to identify subgroups with greater homogeneity compared to preceding studies, with 0 and 73% recurrence rates for grade 1-like and grade 3-like grade 2 subgroups, respectively. We found that gene expression signatures derived using co-expression networks outperform the limited number of genes derived using conventional differential gene expression. Validating this microarray-based classifier with RNA-seq data, we found recurrence rates of 17 and 50% for the same reclassified groups, though the number of samples was insufficient to achieve statistical significance. These findings demonstrate the conceptual advantages of system-based approaches like co-expression networks over conventional techniques like differential gene expression and/or clustering.

### Gene Modules

Interestingly, the modules found to be most predictive of recurrence map to very broad and non-specific molecular functions (RNA splicing and nucleotide synthesis). While this makes the traditional identification of targetable pathways difficult, these domains have been shown to be reliably affected in cancer. Furthermore, they may be targetable with agents such as small molecule splicing modulators and drugs such as rapamycin, respectively (50, 51). We therefore propose further investigation into these sub-disciplines of oncology within the context of meningioma, though these findings remain preliminary and are peripheral to our main findings.

### Limitations

Though our study achieves its purpose, there are a number of limitations which must be considered. Firstly, only a subset of samples have recurrence and follow-up times documented, which may influence generalizability. Furthermore, while a meta-analysis of six independent case-series minimizes bias, there may still be a degree of selection bias as one study is particularly enriched in high grade tumors and the RNA-seq data lacks grade 3 tumors entirely (Table 1). We also acknowledge that the year of WHO grading is not annotated in the data used. However, we consider the grading system used to classify meningiomas in these studies to be post WHO 2007 given that all data were deposited well after 2007. This classification incorporate the updated criteria of the WHO 2000 edition (at least 4 mitoses in 10 high powered fields or 3 of the following criteria: increased cellularity, high nuclear-to-cytoplasm ratios, prominent nucleoli, uninterrupted pattern-less or sheet-like growth, or necrosis) (13). Introduction of these criteria caused a surge in diagnostic rates for grade 2 meningiomas followed by a plateau (13). Importantly, further modifications of the WHO criteria are unlikely to result in increased reporting for grade 2 meningiomas (52), and so the prevalence of meningioma grades in our study is consistent and parallels current practice. Additionally, the objective of this paper is to subdivide grade 2-labeled meningiomas into homogeneous subgroups based on transcriptomics alone, independent of WHO grade, which we have done despite inconsistencies in grade 2 criteria. Finally, our cohort is highly heterogeneous, with patients from geographically diverse centers with potentially different surgical practices and a mixture of microarray and RNA-sequencing platforms. Similarly, a stratification based on relevant mutations in meningioma was not possible due to a lack of sufficient annotation for such an analysis. Nevertheless, we show reclassification of grade 2 meningiomas which is corroborated by the recurrence rates and biological mechanisms which align with the adjacent grade tumor.

### Interpretation

The highly heterogeneous clinical behavior of grade 2 meningiomas suggests that histological criteria do not adequately capture it is biology, thus motivating the segregation into more homogeneous subgroups. So far, molecular profiling of meningiomas has largely taken a monogenetic approach to marker discovery for aggressive phenotypes (22). This has been fruitful in identifying recurrence mutations (15) and transcripts (22) linked to oncogenic cascades in meningiomas. However, these approaches rely on differential gene expression to identify relevant molecular mechanisms and thereby remains limited in its ability to resolve small additive signal often relevant in tumor biology. The use of gene co-expression networks helps to address this limitation. Additionally, a majority of studies on meningioma genetics use histopathological grade as the outcome measure (15), which does not capture disease biology for the case of grade 2 meningiomas. Epigenetic studies using conventional clustering have analyzed heterogeneity of meningiomas across all grades (14) proposing new benign, intermediate and malignant methylation subclasses. “Intermediate” meningiomas are quoted a 20% chance of disease-free survival, which is clinically more useful than the outcome prediction yielded by histology (50%). We believe this study adds to this developing literature surrounding meningioma classification.

### Generalizability

The generalizability of our study is augmented by its design as a meta-analysis, though its purpose is one of hypothesis generation for subsequent, confirmatory studies. Our results therefore require prospective verification and could ultimately help guide molecular diagnostics and prognostics in grade 2 meningiomas. This may ultimately inform recruitment protocols for future and ongoing clinical trials, which are currently limited by the uncertainty of clinical outcomes in grade 2 meningiomas (18). The approach in this study lend to the utility of complex molecular signatures in augmenting histological diagnosis and resolving other heterogeneous and challenging diseases.

## Conclusion

Our findings help resolve the heterogeneity of grade 2 meningiomas by deconvolving them into subgroups which are more homogenous than are proposed in prior studies. These subgroups may help predict clinical course, thus allowing for customized follow-up planning to manage resource intense investigations such serial imaging while optimizing patient care.

## Data Availability Statement

Publicly available datasets were analyzed in this study. This data can be found here: https://www.ncbi.nlm.nih.gov/geo/; accession GSE100534, GSE77259, GSE54934, GSE43290, GSE16581, GSE74385, and GSE136661.

## Ethics Statement

Ethical review and approval was not required for the study on human participants in accordance with the local legislation and institutional requirements. Written informed consent for participation was not required for this study in accordance with the national legislation and the institutional requirements.

## Author Contributions

ZZ conceived the study. ZZ, AL, and AS carried out the analysis. ZZ and AL prepared the figures, wrote the manuscript. MC, ZZ, and AL revised the manuscript. All authors contributed to the article and approved the submitted version.

## Conflict of Interest

The authors declare that the research was conducted in the absence of any commercial or financial relationships that could be construed as a potential conflict of interest.

## Supplementary Material

FIGURE S1 Validating meta-genes as representations of module gene expression. **(A)** The proportion of variance explained by each principle component (PC) in the “tan” (left) and “royal blue” (right) modules. In both cases, the first principle component explains considerably greater variance than any higher PC. **(B,C)** Scatter plot of patients by first and second PCs, with colors representing batch and sex, respectively. The “tan” module is depicted on the left and the “royal blue” module on the right. Only patients with annotated sex are included in **(C)**. Notably, there is no clustering of batch and/or sex evident along the first (or second) PC.

FIGURE S2 Distribution of histopathologic subtypes in the microarray cohort, by individual study. Top bar represents WHO grade (green = 1, black = 2, red = 3). NOS, not otherwise specified.

TABLE S1 Differentially regulated genes between grades 1 and 3.
