# Supplementary figures and images for: Gene Expression Signatures Identify Biologically Homogenous Subgroups of Grade 2 Meningiomas
Source: Front Oncol. 2020 Nov 5;10:541928. doi: 10.3389/fonc.2020.541928 (PMC7674612; doi:10.3389/fonc.2020.541928)

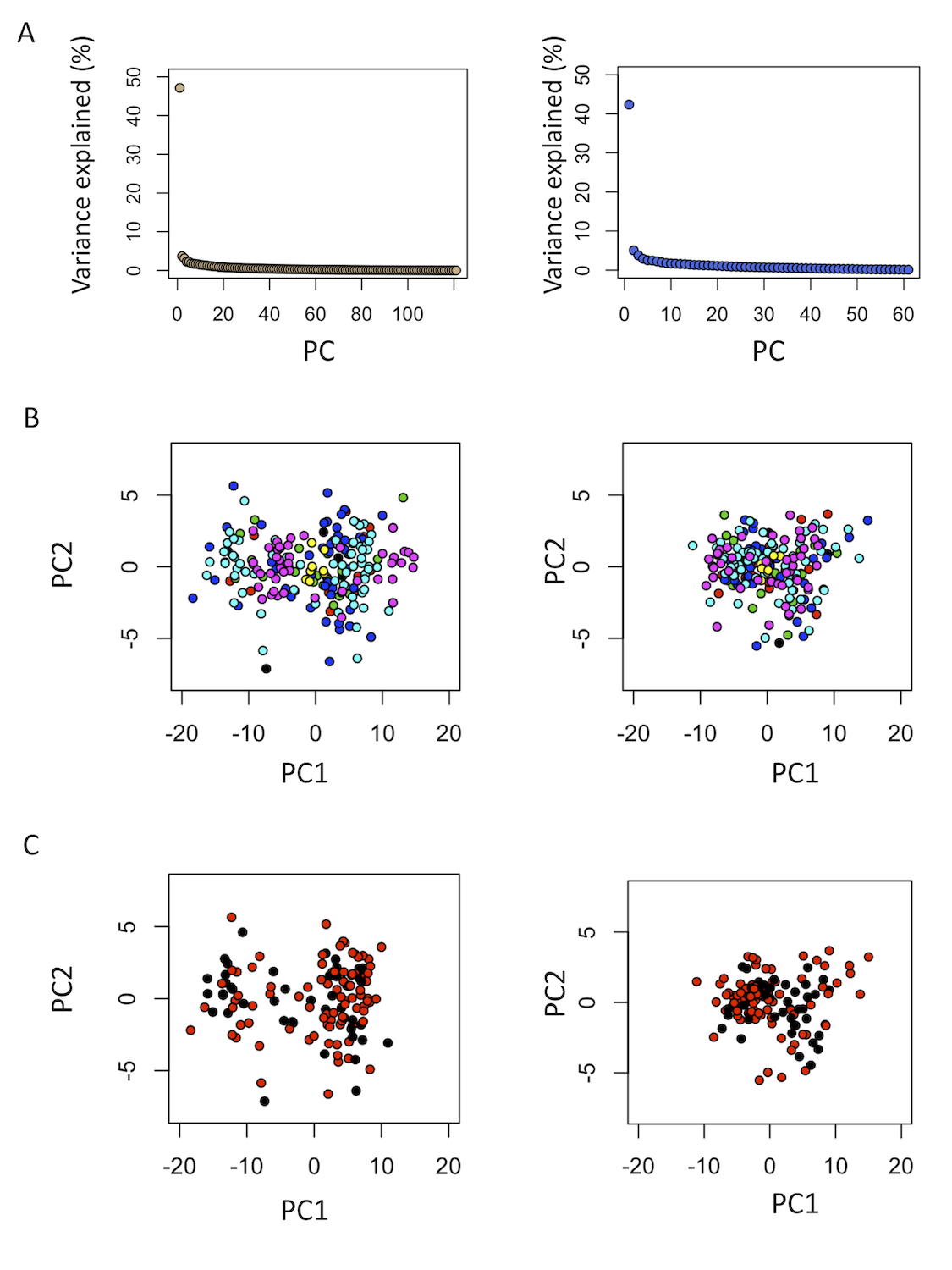

Supplement: FIGURE S1 — Validating meta-genes as representations of module gene expression. (A) The proportion of variance explained by each principle component (PC) in the “tan” (left) and “royal blue” (right) modules. In both cases, the first principle component explains considerably greater variance than any higher PC. (B,C) Scatter plot of patients by first and second PCs, with colors representing batch and sex, respectively. The “tan” module is depicted on the left and the “royal blue” module on the right. Only patients with annotated sex are included in (C). Notably, there is no clustering of batch and/or sex evident along the first (or second) PC. [file Image_1.TIFF]

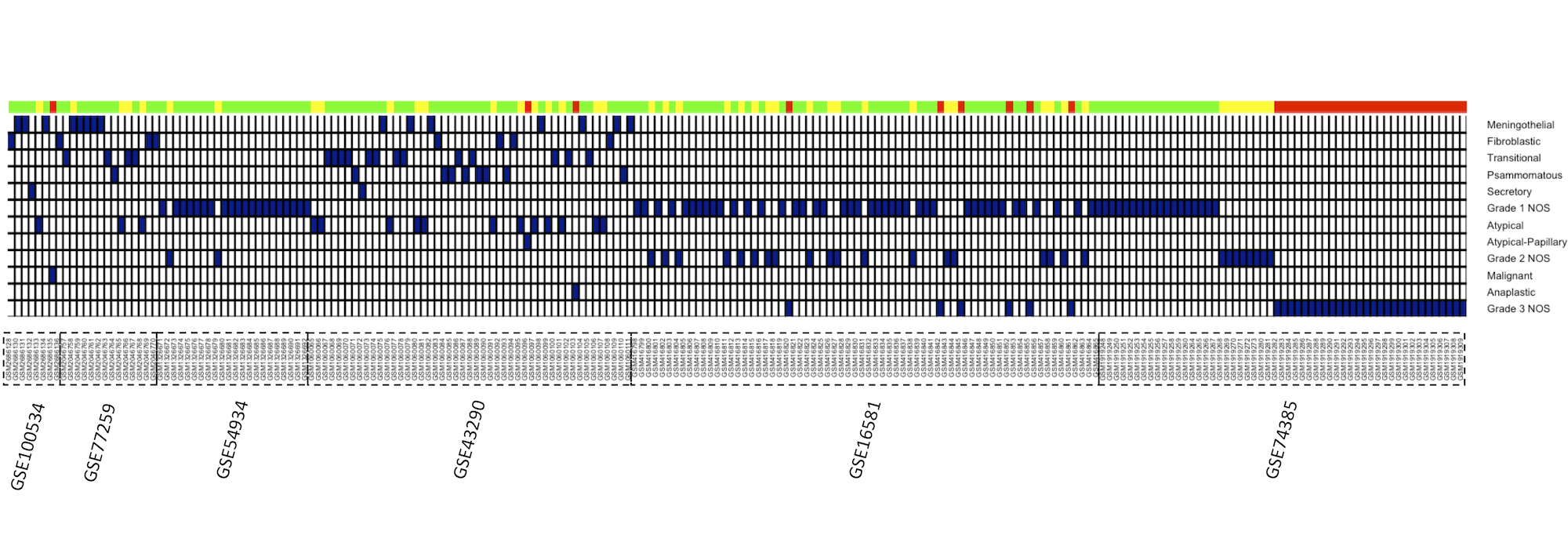

Supplement: FIGURE S2 — Distribution of histopathologic subtypes in the microarray cohort, by individual study. Top bar represents WHO grade (green = 1, black = 2, red = 3). NOS, not otherwise specified. [file Image_2.TIFF]
