# Supplementary material for: Gene Expression Signatures Identify Biologically Homogenous Subgroups of Grade 2 Meningiomas
Source: Front Oncol. 2020 Nov 5;10:541928. doi: 10.3389/fonc.2020.541928 (PMC7674612; doi:10.3389/fonc.2020.541928)
Supplement: TABLE S1 — Differentially regulated genes between grades 1 and 3. [file Data_Sheet_2.PDF]

Supplemental Table 1: Differentially regulated genes between grades 1 and 3.

| Gene Symbol | Gene name | $\log_2$(fold) | $-\log_{10}$(p) |
|---|---|---|---|
| DTL | Denticleless protein homolog | +1.09 | 6.60 |
| SLC7A1 | High affinity cationic amino acid transporter 1 | +1.04 | 8.25 |
| CENPF | Centromere protein F precursor | +1.03 | 6.55 |
| TPX2 | Targeting protein for Xklp2 | +1.02 | 8.87 |
| LEPR | Leptin receptor | -1.05 | 5.15 |
| EGFL6 | Epidermal growth factor like domain multiple 6 | -1.03 | 6.05 |
